# Follow-up of hypertension in patients with multiple sclerosis

**Published:** 2016-07-06

**Authors:** Seyed Mohammad Baghbanian

**Affiliations:** ^1^ Department of Neurology, School of Medicine, Booalisina Hospital, Mazandaran University of Medical Sciences, Sari, Iran

**Keywords:** Multiple Sclerosis, Hypertension, Follow-up

The prevalence of hypertension is estimated over 10% in the multiple sclerosis (MS) population and increases with age.^1^ In some studies comparing the prevalence of hypertension in the MS population with a comparator, hypertension was reported more commonly among these patients.^2^^-^^6^ Only one cohort study reported the incidence of hypertension over a maximum follow-up of 30 years as 3.73% in patients with MS.^7^

Sometimes, new-onset hypertension could be a presenting sign of an adverse event. Transient hypertension may be an adverse event of intravenous methylprednisolone. Hypotension, a known adverse effect of interferon (INF), is a known risk factor of ischemic colitis and ischemic colitis is one of the serious adverse events of treatment with IFNs type I. Ischemic colitis should be considered in INF and acetylcholine inhibitors (AChI) and calcium channel blockers (CCB) co-administration.^8^^,^^9^

Treatment with IFN type I could predispose the patient to develop an autoimmune disease.^10^

Some reports define INF-induced de novo Raynaud’s phenomenon, sometimes with progression to systemic sclerosis. A new-onset accelerated arterial hypertension could be a part of systemic sclerosis triad.^11^ Similarly, new-onset hypertension could be a sign of INF-induced systemic lupus erythematosus (SLE).^12^

Thrombotic microangiopathy is a known rare adverse event of INF-therapy and new-onset hypertension is one of its important presentations advised to be evaluated carefully and controlled regularly in patients with MS receiving IFN-β.^13^

Hypertension is reported in approximately 10% of patients with MS exposed to glatiramer acetate in premarketing studies. During post marketing period, there are reports of hypertensive crisis with glatiramer acetate complicated with acute pulmonary edema and myocardial ischemic injury.^14^

Fingolimod could cause vasodilation and associated hypotension via activation of the endothelial nitric oxide synthase/nitric oxide (eNOS/NO) pathway.^15^^-^^18^ As a result, in some patients experiencing a slight transient hypotension after the initiation of fingolimod therapy, it is not strange. Sometimes, this is followed by a small hypertension (~3 mmHg systolic and ~1 mm Hg diastolic blood pressure); but after 6 months of treatment, hypertension is placed in a stable plateau level.^19^

After the infusion of natalizumab and typically following two days, there are some reports of hypertension but much less frequent; this side effect is defined as probable and very likely.^20^

In teriflunomide trials, hypertension is reported in 3.1 and 4.3% of the patients treated with 7 or 14 mg of teriflunomide compared with 1.8% for the placebo.^21^ In a phase-II teriflunomide clinical trial, high blood pressure was a cause of withdraw.^22^ European medical agency recommends careful hypertension history taking and appropriate management during the treatment with teriflunomide.^23^ Hypertension could be a common side effect of alemtuzumab.^24^

Up to now, there is not any information on arterial hypertension induced by dimethyl fumarate.

Essential hypertension is common in patients with MS similar to general population and probably could affect mortality, morbidity and final disability. New-onset hypertension could be a presenting sign of a treatment adverse event. MS healthcare professionals should measure and observe patients’ blood pressure in follow-up visits and manage it appropriately.

## Notes:

**How to cite this article:** Baghbanian SM. Follow-up of hypertension in patients with multiple sclerosis. Iran J Neurol 2016; 15(3): 180-1.
